# Morphology–Dependent Electrochemical Sensing Properties of Iron Oxide–Graphene Oxide Nanohybrids for Dopamine and Uric Acid

**DOI:** 10.3390/nano9060835

**Published:** 2019-06-01

**Authors:** Zhaotian Cai, Yabing Ye, Xuan Wan, Jun Liu, Shihui Yang, Yonghui Xia, Guangli Li, Quanguo He

**Affiliations:** 1 Hunan Key Laboratory of Biomedical Nanomaterials and Devices, College of Life Sciences and Chemistry, Hunan University of Technology, Zhuzhou 412007, China; caizhaotian1998@163.com (Z.C.); yyb980501@163.com (Y.Y.); wanxuan1111@163.com (X.W.); liu.jun.1015@163.com (J.L.); yangshihui0522@163.com (S.Y.); 2 Zhuzhou Institute for Food and Drug Control, Zhuzhou 412000, China; Sunnyxia0710@163.com

**Keywords:** morphology-dependent, α-Fe_2_O_3_ nanoparticles, GO, uric acid, dopamine, voltammetric detection

## Abstract

Various morphologies of iron oxide nanoparticles (Fe_2_O_3_ NPs), including cubic, thorhombic and discal shapes were synthesized by a facile meta-ion mediated hydrothermal route. To further improve the electrochemical sensing properties, discal Fe_2_O_3_ NPs with the highest electrocatalytic activity were coupled with graphene oxide (GO) nanosheets. The surface morphology, microstructures and electrochemical properties of the obtained Fe_2_O_3_ NPs and Fe_2_O_3_/GO nanohybrids were characterized by scanning electron microscopy (SEM), X-ray diffraction (XRD), cyclic voltammetry (CV) and electrochemical impedance spectroscopy (EIS) techniques. As expected, the electrochemical performances were found to be highly related to morphology. The discal Fe_2_O_3_ NPs coupled with GO showed remarkable electrocatalytic activity toward the oxidation of dopamine (DA) and uric acid (UA), due to their excellent synergistic effect. The electrochemical responses of both DA and UA were linear to their concentrations in the ranges of 0.02–10 μM and 10–100 μM, with very low limits of detection (LOD) of 3.2 nM and 2.5 nM for DA and UA, respectively. Moreover, the d-Fe_2_O_3_/GO nanohybrids showed good selectivity and reproducibility. The proposed d-Fe_2_O_3_/GO/GCE realized the simultaneous detection of DA and UA in human serum and urine samples with satisfactory recoveries.

## 1. Introduction

Dopamine (DA) and uric acid (UA) usually coexist in the serum and extracellular fluids of the central nervous system and play a significant role in regulating human metabolism activity [1]. As an indispensable catecholamine neurotransmitter, DA plays critical roles in regulating the function of the cardiovascular and central nervous systems, as well as maintaining emotional control and hormonal balance [2]. Abnormal levels of DA can lead to various neurological disorders such as schizophrenia, Parkinson’s and Alzheimer’s disease [3,4,5]. Therefore, the precise detection of the DA level in physiological fluids is essential for the early diagnosis of these neurological disorders. However, the rapid and reliable detection of DA in physiological samples remains critical and challenging due to the low DA concentration in the extracellular matrix (usually in the range of 0.01–1 μM) and its susceptibility to interference from endogenous substances such as UA and ascorbic acid (AA). Uric acid is another crucial biomolecule in physiological fluids and is often regarded as the end-product of purine metabolism in the human body [6]. For a heathy individual, the UA concentration is generally in the range of 4.1 ± 8.8 mg/100 mL [7]. The dysfunction of UA in bodily fluids likely causes several diseases, including gout, pneumonia and hyperuricemia [8]. As stated, both DA and UA are regarded as important biomolecules for the regulation of human metabolic activity. Thus, determining the concentration of DA and UA in biological matrices (i.e., human urine, serum) can provide valuable clues for healthcare and disease diagnosis. Since DA and UA usually coexist in physiological fluids, it is of the utmost importance to propose a highly efficient technique for the simultaneous determination of DA and UA.

To date, several analytical techniques have been proposed for the detection of DA and UA, including, but not limited to, high performance liquid chromatography [9,10], fluorescent [11], spectrophotometry [12], electrogenerated chemiluminescent [13] and surface plasmon resonance [14] etc. Although quite reliable, these techniques usually involve tedious and time-consuming analytical procedures that require expensive equipment, well-trained technical personnel or a large quantity of toxic solvents [15]. Compared with other techniques, the electroanalytical techniques are more suitable for sensing DA and UA because of their low price, fast response, facile operation and excellent anti-interfering ability [16,17,18]. Owing to the considerable superiorities including cost-effectiveness, rapidness, convenience and high efficiency, electroanalytical methods have drawn increasing attention for the detection of small biomolecules, food additives and contaminants [19,20,21,22,23,24]. DA and UA are electroactive species whose redox processes can be quantitatively detected by electroanalytical techniques. However, it becomes a great challenge to simultaneously detect DA and UA on bare electrodes due to the fouling effect that occurs during the oxidation [7] and cross-interferences as a result of similar oxidation potentials [25]. To resolve this problem, nanostructured materials were employed to achieve high sensitivity and prevent overlapping of the oxidation peaks [25]. In recent years, much effort has been devoted to developing promising alternatives as sensing materials for the simultaneous detection of DA and UA, including noble metal nanoparticles [25], metal oxides nanocomposites [26], alloyed nanoparticles [27], polymer films [28,29], and nanocarbon materials [30,31,32] etc.

Iron oxide (Fe_2_O_3_) has become one of the most versatile transition metal oxides not only due to low cost, more abundant, good biocompatibility and excellent electrochemical performances, but also for its widespread applications [33,34,35,36,37,38]. In particular, α-Fe_2_O_3_ nanoparticles (α-Fe_2_O_3_ NPs) have been considered as the most promising modifying material, because of the variable valence state of iron oxides that can be recovered in situ via electrochemical reducing or oxidizing during the sensing process, thus triggering the heterogeneous redox of the target analysts [39]. As far as we know, various morphologies of α-Fe_2_O_3_ NPs have been made available in previous reports, including wire [40], rod [41,42], tube [41], sphere [43,44], flower [45], spindle [44], cubic [44,46,47,48,49], thorhombic [46,47,48], discal [47], and shuttle [50]. Many studies demonstrate that the morphologies of nanostructured α-Fe_2_O_3_ have a significant impact on optical, magnetic, photocatalytic and electrochemical properties [46,47,51,52]. However, the morphology-dependent electrochemical sensing performances with respect to small biomolecules have rarely been investigated. Hence, it is essential to explore the morphology–dependent sensing properties of different α-Fe_2_O_3_ NPs. Unfortunately, the electrochemical sensing performances of pure α-Fe_2_O_3_ NPs modified electrodes are relatively poor probably because of their poor electrical conductivity and dispersibility [37,53,54]. To address this issue, iron oxides were often used in a composite with graphene for the detection of DA. For example, nitrogen and sulfur dual doped graphene-supported Fe_2_O_3_ (NSG-Fe_2_O_3_) has been utilized for the electrochemical detection of DA in the presence of AA, with a wide linear response range (0.3–210 μM) and low LOD (0.035 μM) [55]. However, the procedure for the doping of nitrogen and sulfur is very complicated. Moreover, the simultaneous detection of DA and UA is not clear for this nanocomposite. As an important derivant of graphene, graphene oxide (GO) usually works as a conductive component to enhance the electron transfer between electrode surface and target analysts [18,19]. Indeed, there are abundant hydrophilic oxygen-containing functional groups (OxFGs) presented on the hydrophobic basal planes of GO, which can behave like an amphiphilic surfactant to improve the dispersion of α-Fe_2_O_3_ NPs [56]. As far as we know, α-Fe_2_O_3_/GO nanocomposites have rarely reported for the simultaneous detection of DA and UA.

Herein, α-Fe_2_O_3_ NPs with various morphologies including cubic, thorhombic and discal shapes were synthesized by a facile meta-ion mediated hydrothermal route then composite with GO nanosheets. The electrocatalytic activities of DA and UA at Fe_2_O_3_/GO nanohybrids decorated glassy carbon electrodes (Fe_2_O_3_/GO/GCE) were measured in this work. The electrochemical measurements exhibited that the discal Fe_2_O_3_ NPs had the most remarkable electrochemical response toward the simultaneous detection of DA and UA, attributing to the more surface defects and rougher surface. After further coupled with GO, the discal α-Fe_2_O_3_ NPs/GO nanohybrids (d-Fe_2_O_3_/GO) showed superior electrochemical sensing performances toward DA and UA, due to the notable synergistic effect from d-Fe_2_O_3_ and GO. Therefore, a novel and ultrasensitive electrochemical sensor based on d-Fe_2_O_3_/GO nanohybrids was proposed for the simultaneous detection of DA and UA.

## 2. Materials and Methods

### 2.1. Chemicas and Solutions

Dopamine (DA), uric acid (UA), Iron (III) nitrate nonahydrate (Fe(NO_3_)_3_·9H_2_O), cupric acetate anhydrous (Cu(Ac)_2_), Zinc acetate (Zn(Ac)_2_·2H_2_O), aluminum acetate (Al(Ac)_3_), disodium hydrogen phosphate dodecahydrate (Na_2_HPO_4_·12H_2_O) and Sodium dihydrogen phosphate dihydrate (NaH_2_PO_4_·2H_2_O) were purchased from Aladdin Reagents Co., Ltd. (Shanghai, China). Graphite powder, potassium permanganate (KMnO_4_), hydrogen peroxide (H_2_O_2_, 30%), sodium nitrate (NaNO_3_), potassium ferricyanide(K_3_[Fe(CN)_6_]), potassium ferrocyanide (K_4_[Fe(CN)_6_]), ammonia (NH_3_·H_2_O), sodium hydrate (NaOH), concentrated hydrochloric acid (HCl, 37%), concentrated sulfuric acid (H_2_SO_4_, 98%) and absolute ethanol (CH_3_CH_2_OH) were supplied by Sinopharm Chemical Reagent Co. Ltd. (Shanghai, China). All chemicals were analytically pure and directly used as received. Human serum samples were provided by Zhuzhou People’s Hospital (Zhuzhou, China). The stock solutions of DA and UA (1 mM) were prepared by dissolving appropriate amount of DA and UA in 500 mL 0.1 M PBS. Then lower concentration series of the standard solution were obtained by appropriately diluting the stock solution with 0.1 M PBS. Deionized water (DI water, 18.2 MΩ) was used in all the experiments.

### 2.2. Apparatus

All the electrochemical measurements were performed on a CHI 760E electrochemical workstation (Chenhua Instrument Inc., Shanghai, China). Surface morphologies and crystalline structure of α-Fe_2_O_3_ NPs, and Fe_2_O_3_/GO were investigated by scanning electron microscopy (SEM) and powder X-ray diffractometry (XRD), respectively. SEM images were recorded on a cold field-emission SEM (Hitachi S-4800, Tokyo, Japan) at an accelerating voltage of 5.0 kV. The XRD patterns of α-Fe_2_O_3_ NPs was recorded using a powder X-ray diffractometer system (PANalytical, Holland) with monochromatized Cu Kα radiation (λ = 0.1542 nm) operating at 40 kV and 40 mA. The patterns were collected in a 2θ range from 10° to 80° with a scanning step of 0.02°2θ s^−1^. A digital precision pH meter (Leici instrument Inc., Shanghai, China) was used to measure solution pH.

### 2.3. Synthesis of Discal, Thorhombic, and Cubic α-Fe_2_O_3_ NPs

**Scheme 1:**
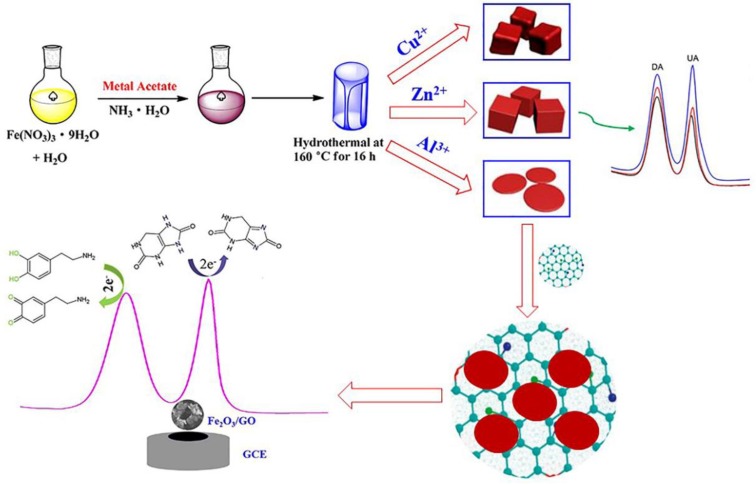
The schematic illustration of meta-ion mediated hydrothermal route for the synthesis of discal, thorhombic, and cubic α-Fe_2_O_3_ NPs and possible mechanism for the electrochemical sensing of DA and UA at the d-Fe_2_O_3_/GO/GCE.

Referring to previous reports [46,47,48], three types of α-Fe_2_O_3_ NP_S_ were synthesized by a meta-ion mediated hydrothermal route (Scheme 1). In briefly, 0.808 g of Fe(NO_3_)_3_·9H_2_O was adequately dissolved into 10 mL of DI water under magnetic stirring, and then 1 mM of acetate precursor was added to the above solution. After 15 min of stirring, 10 mL NH_3_·H_2_O was added and continuously stirring for another 15 min. Next, the mixture was poured into a 50 mL Teflon-lined stainless-steel autoclave and reacted at 160 °C for 16 h. Subsequently, the autoclave was naturally cooled down to room temperature. Finally, the resultant α-Fe_2_O_3_ product was alternately washed three times with absolute alcohol and DI water, and allowed to dry under vacuum at 60 °C for 12 h. Herein, cupric acetate, zinc acetate, and aluminum acetate were separately used as acetate precursor to obtain thorhombic, cubic and discal α-Fe_2_O_3_ NPs.

### 2.4. Preparation of Fe_2_O_3_/GO Composite

GO was synthesized by a slightly modified Hummer’s method according to our earlier reports [20,23,24]. 10 mg GO was delaminated and dispersed in the 10 mL DI water under ultrasonication for 2 h to form 1 mg/mL GO dispersion. Then one aliquot of as-obtained α-Fe_2_O_3_ products was added into five aliquots of GO dispersion (1 mg/mL), and then ultrasonicated for 0.5 h to form uniform α-Fe_2_O_3_/GO dispersion.

### 2.5. Fabrication of Fe_2_O_3_/GO/GCE

At first, bare GCE (ca. 0.0707 cm^2^) was carefully polished to mirror-like with 0.3 μm and 0.05 μm fine alumina slurry. Then the polished GCE was alternately washed by absolute ethanol and DI water several times and allowed to dry under an infrared lamp. The Fe_2_O_3_/GO/GCE was prepared by a simple drop-casting method. More specifically, 5 μL Fe_2_O_3_/GO dispersion was carefully dropped and coated on the GCE surface with a micropipette, then dried with an infrared lamp to form sensing film. For comparison, the discal, thorhombic, and cubic α-Fe_2_O_3_ NPs decorated GCEs (d-Fe_2_O_3_/GCE, t-Fe_2_O_3_/GCE, c-Fe_2_O_3_/GCE) and GO decorated GCE (GO/GCE) were also fabricated by similar procedure.

### 2.6. Electrochemical Measurements

The electrochemical behaviors of various decorated electrodes were measured by cyclic voltammetry (CV) and electrochemical impedance spectroscopy (EIS) in the 0.1 M PBS (pH 7.0) containing 0.5 mM [Fe(CN)_6_]^3−/4−^ as redox probe solution. EIS was measured at the frequency range from 100 kHz to 0.1 Hz at open circuit potential with 5 mV amplitude, using a redox couple of 0.5 mM [Fe(CN)_6_]^3−/4−^ (1:1) in 0.1 M KCl as an electrochemical probe. The electrochemical responses of 10 μM DA and UA (1:1) mixture standard solution at different electrodes were recorded by CV. Differential pulse voltammetry (DPV) was used for the individual and simultaneous detection of DA and UA. A conventional three-electrode assembly was immersed into a 10 mL electrochemical cell for electrochemical measurements and detections, consisting of a bare or modified GCE as the working electrode, a saturated calomel electrode (SCE) as the reference electrode, and a platinum wire as auxiliary electrode, respectively. To increase response peak current, a suitable accumulation period was applied before CV and DPV measurements. DPV was performed from 0 V to 0.8 V, with step potential of 4 mV, the amplitude of 50 mV and pulse width of 0.06 s and pulse period of 0.5 s, respectively. 0.1 M PBS was used as the supporting electrolytes unless otherwise specified. All the electrochemical measurements were performed at 25 °C.

## 3. Results and Discussions

### 3.1. Morphology–Dependent Electrochemical Sensing of α-Fe_2_O_3_ NPs

**Figure 1:**
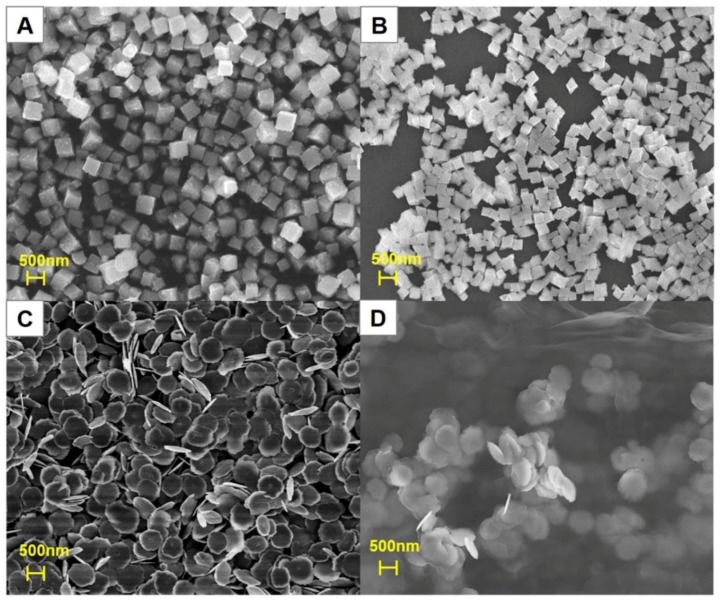
Scanning electron microscopy (SEM) images of α-Fe_2_O_3_ NPs with cubic (**A**), thorhombic (**B**) and discal (**C**) morphology, discal α-Fe_2_O_3_ NPs/GO nanohybrid (**D**).

**Figure 2:**
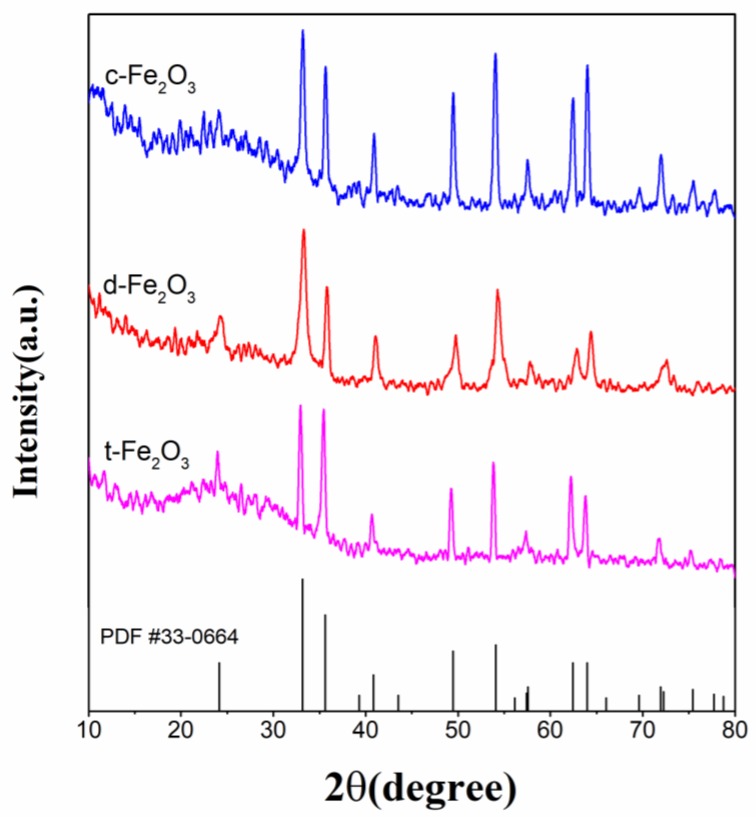
X-ray diffraction (XRD) patterns of α-Fe_2_O_3_ NPs with cubic (c-Fe_2_O_3_), discal (d-Fe_2_O_3_), and thorhombic (t-Fe_2_O_3_) morphologies.

The schematic diagram of the preparation of the α-Fe_2_O_3_ NPs is shown in Scheme 1. The α-Fe_2_O_3_ NPs with cubic (c-Fe_2_O_3_ NPs), discal (d-Fe_2_O_3_ NPs), and thorhombic (t-Fe_2_O_3_ NPs) shapes were prepared via different metal-ion mediated hydrothermal treatment [46,47,48]. The α-Fe_2_O_3_ NPs are produced as cubic, discal and thorhombic shapes, due to the addition of Zn^2+^, Al^3+^, and Cu^2+^ ions, respectively. Figure 1A–C shows the SEM images of as-obtained α-Fe_2_O_3_ NPs. Clearly, three α-Fe_2_O_3_ NPs with uniform cubic, thorhombic and discal shapes are observed, suggesting uniform cubic, thorhombic and discal shapes were successfully prepared. The crystalline structures of α-Fe_2_O_3_ NPs with three distinct morphologies were further investigated by XRD. As presented in Figure 2, the diffraction peak*s* in all the patterns can be well assigned to the rhombohedral phase of hematite (JCPDS PDF# 33-0664) [46]. Moreover, no apparent diffraction peaks relating to CuFe_2_O_4_, ZnFe_2_O_4_ and AlFeO_3_ can be found, demonstrating the crystalline structures of the α-Fe_2_O_3_ NPs are not altered by the addition of Cu^2+^, Zn^2+^ and Al^3+^ ions.

**Figure 3:**
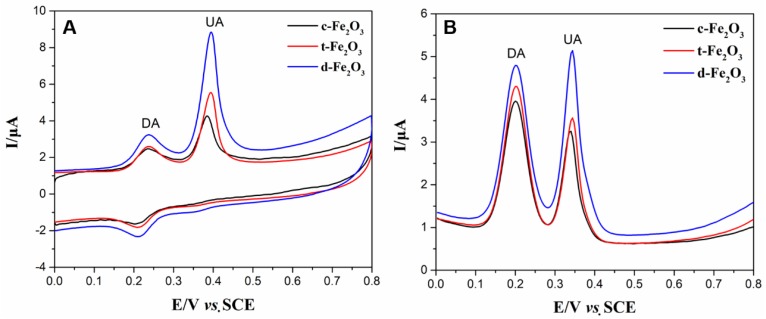
Cyclic voltammetry (CV) (**A**) and differential pulse voltammetry (DPV) (**B**) curves of c-Fe_2_O_3_, t-Fe_2_O_3_, and d-Fe_2_O_3_ NPs modified GCEs in 0.1 M PBS (pH = 7) containing 10 μM DA and UA.

For comparison purposes, α-Fe_2_O_3_ NPs with various morphologies were utilized for the simultaneous detection of 10 μM DA and UA in 0.1 M PBS through CV and DPV technique, and the results are shown in Figure 3A,B, respectively. On all the electrodes, two anodic peaks for DA and UA are independent and evident (Figure 3A), suggesting high selectivity. However, a very small cathodic peaks relating to UA are observed on all the electrode at reverse scanning, demonstrating the reversibility of UA redox is very poor. Note that a pair of redox peaks can be observed on all the electrodes with *I*_pa_/*I*_pc_ ranging from 1.08 to 1.39, indicating that DA undergoes a quasi-reversible electrode process. The increased anodic peak heights in the CVs indicates enhanced electrocatalytic activity. Among the three morphologies of α-Fe_2_O_3_ NPs modified electrodes, d-Fe_2_O_3_/GCE exhibits highest electrocatalytic activity toward DA and UA, with well-shaped anodic peaks (*I*_pa(DA)_ = 0.1764 μA, *I*_pc(UA)_ = 0.6626 μA) appear at 0.238 and 0.395 V for DA and UA, respectively. Compared to c-Fe_2_O_3_/GCE and t-Fe_2_O_3_/GCE, the anodic peak current for DA at d-Fe_2_O_3_/GCE increases by 65.8% and 30.0% and the anodic peak current for UA increases by 177% and 73.1%, respectively. Liu et al. systematically compared the photocatalytic activity among cubic, discal and thorhombic α-Fe_2_O_3_ NPs, d-Fe_2_O_3_ NPs were found to have more surface defects and rougher surface which result in highest photocatalytic activity [47]. Generally, the electrochemical active sites will be more accessible on the more defective surface, which can greatly facilitate the electrocatalytic oxidation of target analysts. Moreover, the rougher surface means a larger specific surface area, which is also favorable for electrochemical sensing. These facts explain well the remarkable electrocatalytic activity for d-Fe_2_O_3_. For the DPVs in Figure 3B further confirmed discal α-Fe_2_O_3_ NPs have the strongest electrocatalytic ability for the oxidation of DA and UA. Therefore, there is solid proof that the electrochemical activity of α-Fe_2_O_3_ NPs can be tailored by their morphologies. Considering the excellent sensitivity for the simultaneous determination of DA and UA, high electrocatalytic active discal α-Fe_2_O_3_ NPs were chosen as sensing materials in subsequent experiments. To further enhance the sensitivity, discal α-Fe_2_O_3_ NPs were coupled with GO (d-Fe_2_O_3_/GO) aiming to yield synergistic effect toward the electrooxidation of DA and UA. The SEM image of d-Fe_2_O_3_/GO is shown in Figure 1D. Evidently, discal α-Fe_2_O_3_ NPs are warped by silky graphene oxide sheets.

### 3.2. Electrochemical Performance of d-Fe_2_O_3_/GO/GCE

**Figure 4:**
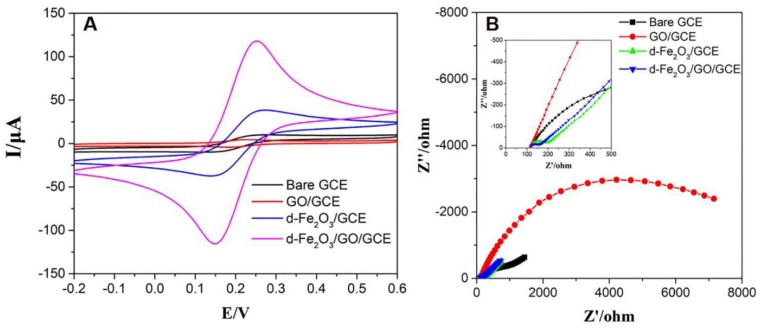
(**A**) CVs curves of bare GCE, GO/GCE, d-Fe_2_O_3_/GCE and d-Fe_2_O_3_/GO/GCE in the 0.5 mM [Fe(CN)_6_]^3−/4−^; (**B**) Nyquist plots of different electrodes for the EIS measurements in the presence of 0.5 mM [Fe(CN)_6_] ^3−/4−^ (1:1) in 0.1 M KCl. The inset is the magnification of Nyquist plots at the higher frequency region.

The electrochemical performance of different modified GCEs were investigated by CV using 0.5 mM [Fe(CN)_6_]^3−/4−^ as probe solution. The corresponding CV curves are plotted in Figure 4. A pair of weak redox peaks (*I*_pa_ = 12.73 μA, *I*_pc_ = 12.16 μA) are observed on the bare GCE with peak potential separation (∆*E*_p_) of 0.156 V, suggesting that the electron transfer rate is very slow. At the GO/GCE, a pair of wide and weak redox peak occurs with the lowest redox peak currents (*I*_pa_ = 3.942 μA, *I*_pc_ = 3.768 μA) and maximum ∆*E*_p_ (∆*E*_p_ = 0.182 V). It was mainly due to the presence of poor electrical conductivity of GO. At the d-Fe_2_O_3_/GCE, a pair of well-shaped and sharp redox peak appears. Both anodic peak current (*I*_pa_) and cathodic peak current (*I*_pc_) increase greatly (*I*_pa_ = 3.942 μA, *I*_pc_ = 3.768 μA) while the ∆*E*_p_ decreases to 0.136 V, indicating d-Fe_2_O_3_ can effectively promote the redox process due to its high electrocatalytic activity. As expected, d-Fe_2_O_3_/GO/GCE displays remarkable electrochemical properties with the highest redox peak currents (*I*_pa_ = 47.45 μA, *I*_pc_ = 46.8 μA) and minimum potential separation (∆*E*_p_ = 0.104 V), attributing to the synergistic effect from d-Fe_2_O_3_ NPs and GO nanosheets. As well known, electrochemical active area is highly related to the electrochemical sensing properties. The electrochemical active areas of various electrodes were also estimated using the Randles-Sevcik equation [20,21,24]. The electrochemical active areas of bare GCE, GO/GCE, d-Fe_2_O_3_/GCE and d-Fe_2_O_3_/GO/GCE are estimated as 0.1037, 0.0321, 0.3991 and 1.1181 cm^2^, respectively. The electrochemical active area of d-Fe_2_O_3_/GO/GCE is approximately 2-fold and 10-fold higher than that of d-Fe_2_O_3_/GCE and bare GCE, respectively.

EIS is a valuable tool to acquire interfacial properties, which has extensively used in the various electrochemical sensors [20,21,57,58,59]. Nyquist plots for bare GCE, d-Fe_2_O_3_/GCE, GO/GCE and d-Fe_2_O_3_/GO/GCE are presented in Figure 4B. The Nyquist plots reveals electron transfer kinetics and diffusion characteristic. Typically, Nyquist plots include two portions, namely semicircular and linear portions. The semicircular at the higher frequency domain and linear part at the lower frequency domain represents the electron transfer limited and diffusion limited electrode processes, respectively. The semicircle diameter indicates the charge transfer resistance (*R*_ct_). Obviously, the largest semicircle diameters (4500 ohm) are obtained at GO/GCE, indicating the poor electrical conductivity of GO retard electron transfer process. Compared to bare GCE (948 ohm), the *R*_ct_ values for d-Fe_2_O_3_/GCE and d-Fe_2_O_3_/GO/GCE decrease to 64 ohm and 35 ohm, respectively. The lowest *R*_ct_ value is obtained at d-Fe_2_O_3_/GO/GCE since numerous electrocatalytic active sites occurred, demonstrating that an obvious acceleration of redox kinetics of [Fe(CN)_6_]^3−/4−^ happened on the surface of d-Fe_2_O_3_/GO nanohybrids. The results verify that the d-Fe_2_O_3_/GO nanohybrids can effectively lower the *R*_ct_.

### 3.3. Electrochemical Behaviors of DA and UA on the d-Fe_2_O_3_/GO/GCE

**Figure 5:**
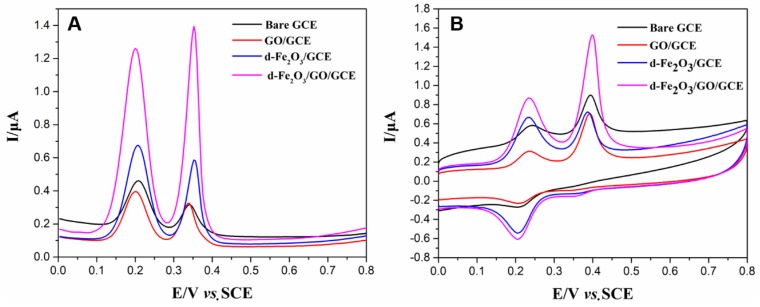
CVs (**A**) and DPVs (**B**) of 10 μM DA and UA (1:1) mixture solution at the bare GCE, GO/GCE, d-Fe_2_O_3_/GCE and d-Fe_2_O_3_/GO/GCE.

Electrochemical responses of 10 μM DA and UA (1:1) at different electrodes were recorded by DPV in 0.1 M PBS (pH = 5.65). As presented in Figure 5A, the anodic peaks of DA and UA are well separated, indicating simultaneous detection of DA and UA is very feasible. On the bare GCE, the *I*_pa_ of DA and UA is 0.2724 μA and 0.1555 μA, respectively. After the modification of GO nanosheets, the *I*_pa_ of DA and UA increased a little (*I*_pa(DA)_ = 0.2915 μA, *I*_pa(UA)_ = 0.2356 μA). The large specific area of GO could provide abundant adsorption sites for target analytes, which enhances the electrochemical response signals. But the poor electrical conductivity of GO severely retards the redox process, which impedes electrochemical oxidation of DA and UA. When d-Fe_2_O_3_ NPs were modified on the GCE, the *I*_pa_ of both DA and UA boosts greatly (*I*_pa(DA)_ = 0.5519 μA, *I*_pa(UA)_ = 0.4770 μA), suggesting d-Fe_2_O_3_ NPs have high electrocatalytic activity toward DA and UA. Among all the electrodes, d-Fe_2_O_3_/GO nanohybrids show a remarkable electrocatalytic capacity toward the electrochemical oxidation of DA and UA, with the highest *I*_pa_ of 1.077 and 1.234 μA for DA and UA, respectively. The synergistic effect from d-Fe_2_O_3_ NPs and GO nanosheets contributed to the significant enhancement in the response currents. Specifically, abundant active sites presented in the GO surface greatly facilities the adsorption of target analystes; d-Fe_2_O_3_ can efficiently catalyzed the electrooxidation of DA and UA. Besides, the d-Fe_2_O_3_ NPs/GO nanohybrids remarkably decrease the electron transfer resistant (confirmed by EIS, Figure 4B), which greatly accelerate the redox kinetics. Moreover, the peak-to-peak separations of bare GCE, GO/GCE, d-Fe_2_O_3_/GCE and d-Fe_2_O_3_/GO/GCE are 0.132, 0.140, 0.144, and 0.148 V, respectively. The maximum ∆*E*_p_ is obtained at d-Fe_2_O_3_/GO/GCE, demonstrating good distinguish capacity.

The electrochemical responses of various electrodes were also measured by CV. As shown in Figure 5B, two anodic peaks belonging to DA and UA are observed at all the electrodes. However, a negligible cathodic peak corresponding to the reduction of UA appears at reverse scanning, implying a poor reversible electrode reaction for UA. The ratio of *I*_pa_/*I*_pc_ approaches 1 for all the electrodes, meaning the electrode undergoes a quasi-reversible process. Among all electrodes, d-Fe_2_O_3_/GO nanohybrids also have largest response peak currents for DA and UA. The CV result is in good agreement with the results obtained by DPVs. It is worth pointing out that DPV is more sensitive than CV, which is very suitable for multiple analystes detection. Therefore, DPV was used for the quantitative analysis of DA and UA.

### 3.4. Optimation of Voltammetric Parameters

#### 3.4.1. Influence of pH

**Figure 6:**
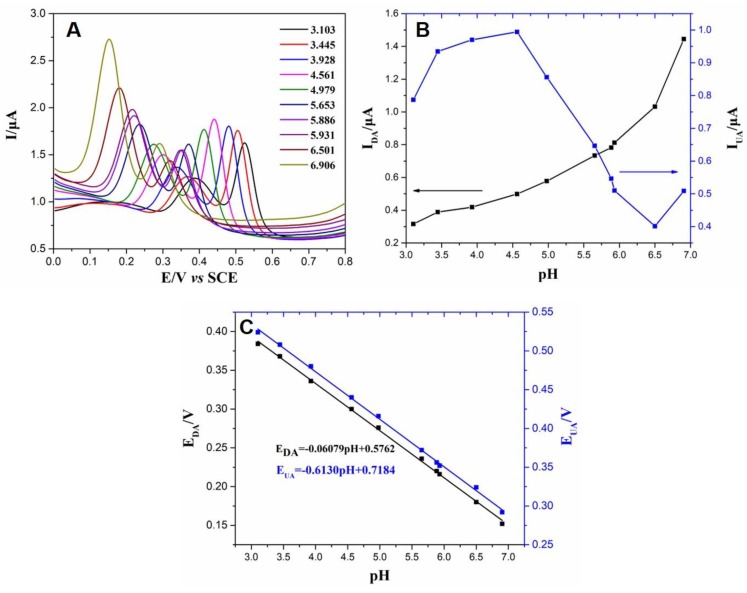
(**A**) DPVs of 10 μM DA and UA (1:1) mixture solution recorded at different pH; (**B**) Effect of pH on the response anodic peak current of DA and UA; (**C**) Linear plots of the anodic peak potential of DA and UA against pH.

It is well known that pH is a vital parameter that directly influences the electrochemical responses. The DPVs of 10 μM DA and UA mixture solution (1:1) at different pH are depicted in Figure 6A. Notably, the anodic peaks shift to the negative direction as the increasing of pH. The influence of pH on the response peak currents of DA and UA is shown in Figure 6B. In the pH range of 3.10 to 6.90, the anodic peak currents always increase with the increase of pH. The anodic peak currents of UA slowly increase when pH varied from 3.10 to 4.56, then gradually decrease with the further increase of pH. The highest response anodic peak is obtained at pH = 4.56. Trading off the oxidation peak current of DA and UA, pH 5.65 is selected as optimum pH in the following measurements. Moreover, the anodic peak potentials linearly decrease with the increase of pH (Figure 6C). The linear plots of peak potential versus pH can be expressed as *E*_DA_ = −0.06079 pH + 0.5762 (*R*^2^ = 0.9988) and *E*_UA_ = −0.06130 pH + 0.7184 (*R*^2^ = 0.9986). The slopes of these linear plots (−60.8 mV/pH and −61.3 mV/pH for DA and UA, respectively) are very close to the theoretical value from the Nernst equation (−59 mV/pH), indicating that the equal numbers of protons (H^+^) and electrons (e^−^) participate in the electrooxidation process of DA and UA, which is in good agreement with previous works.

#### 3.4.2. Influence of Accumulation Parameters

**Figure 7:**
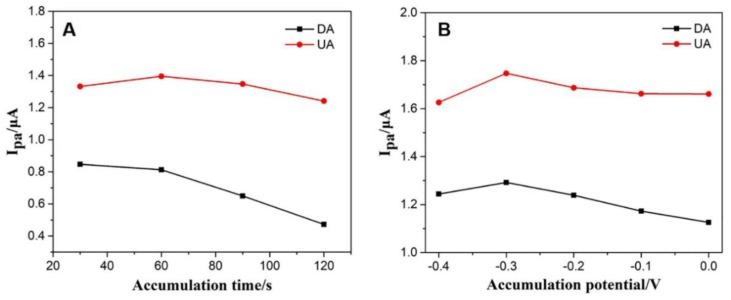
Influence of accumulation time (**A**) and accumulation potential (**B**) on the response anodic peak currents of DA and UA.

To enhance the response electrochemical peak currents, accumulation was applied before electrochemical measurements. The influence of accumulation time on the response anodic peak currents of DA and UA was explored (Figure 7A). The response peak currents of DA and UA slowly increase during the first 60 s, then gradually attenuate with further extending of accumulation time. The highest response peaks are obtained at 60 s. Furthermore, the dependence of the anodic peak currents on the accumulation potential was also investigated (Figure 7B). When accumulation potentials vary from −0.4 to −0.3 V, the anodic peak currents of DA and UA enhance gradually. However, the anodic peak currents decline with accumulation potential varying from −0.3 to 0 V. The maximum anodic peaks are obtained at −0.3 V. Therefore, 60 s and −0.3 V were chosen as the optimum accumulation parameters.

### 3.5. Reaction Mechanism of DA and UA

**Figure 8:**
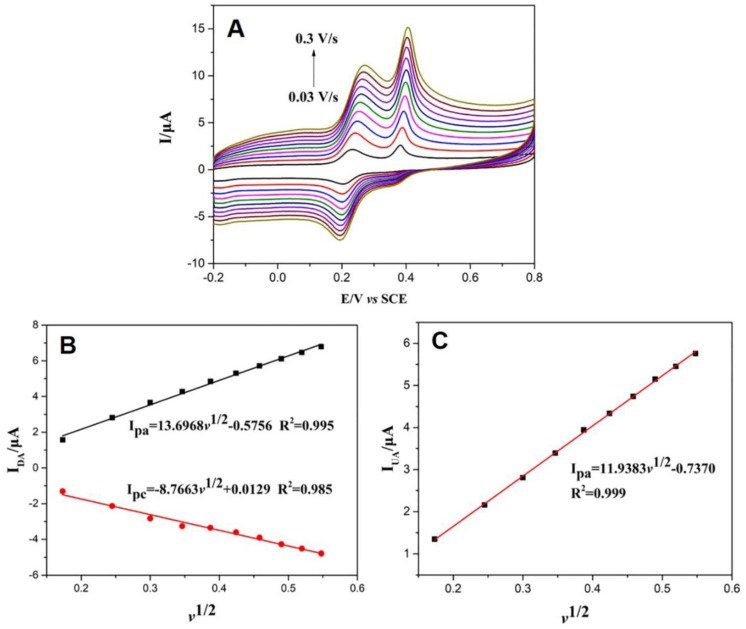
(**A**) CVs of 10 μM DA and UA (1:1) mixture solution recorded at various scanning rates; (**B**) Linear relationship between the redox peak current of DA and the square root of scanning rate (*v*^1/2^); (**C**) Linear relationship between the anodic peak current of UA and the square root of scanning rate (*v*^1/2^).

In order to provide a deeper insight into the electrochemical oxidation mechanism, CVs of 10 μM DA and UA (1:1) mixture solution were recorded at various scanning rates using d-Fe_2_O_3_/GO/GCE. CVs at different scanning rates are plotted in Figure 8A. Notably, the redox peaks increase with the increasing of scanning rate. Meanwhile the background currents also increased, probably because high scanning rates can increase the charging current of double layer. Moreover, the anodic peaks for DA and UA shift positivity as the increase of scanning rate while the cathodic peaks for UA shift to negative direction. As shown in Figure 8B, the anodic and cathodic peak currents of DA are highly correlated with the square root of scanning rate (*v*^1/2^), suggesting the electrooxidation of DA is a diffusion-limited process. The linear regression equations are *I*_pa(DA)_ = 13.6968 *v*^1/2^ −0.5756 and *I*_pc(DA)_ = −8.7663 *v*^1/2^ + 0.0129, with the correlation coefficient (*R*^2^) of 0.985. The anodic peak currents of UA are in positive proportion to the square root of scanning rate (*v*^1/2^), indicating the oxidation of UA limited by the diffusion in bulk solution. The linear regression equation is *I*_pa(UA)_ = 11.9383 *v*^1/2^ − 0.7370 with the correlation coefficient (*R*^2^) of 0.999 (Figure 8C). Since the electrooxidation of DA and UA is an equal number of electrons (e^−^) coupled with protons (H^+^) process, a two electron and two proton (2H^+^, 2e^−^) mechanism was proposed for the oxidation of DA and UA (Scheme 1).

### 3.6. Individual and Simultaneous Detection of DA and UA

**Figure 9:**
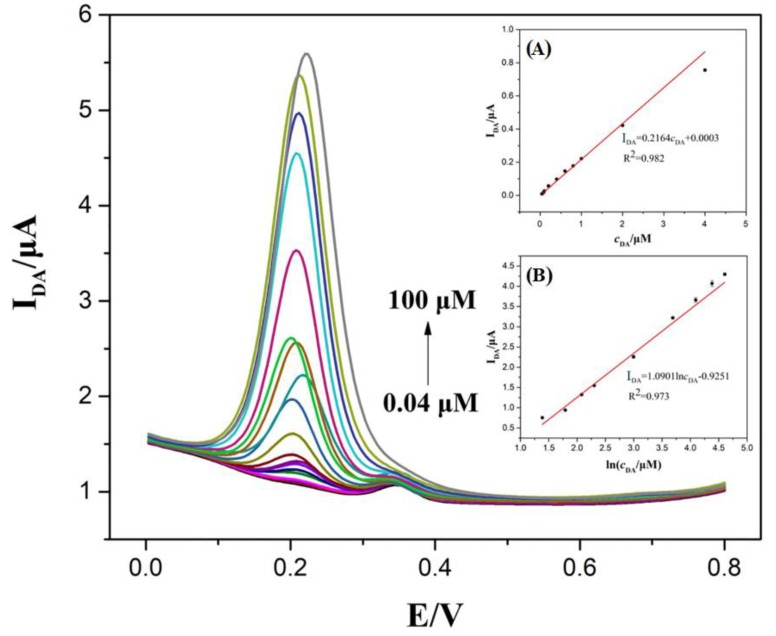
DPVs on the d-Fe_2_O_3_/GO/GCE in 0.1 M PBS (pH 5.65) containing 1 μM UA and various concentrations of DA from 0.04 to 100 μM; The inset (**A**) represents the linear plot of the anodic peak currents versus the DA concentrations varying from 0.04 to 4 μM; The inset (**B**) represents the linear plot of the anodic peak currents versus the Napierian logarithm of DA concentrations with DA concentration ranging from 4 to 100 μM.

**Figure 10:**
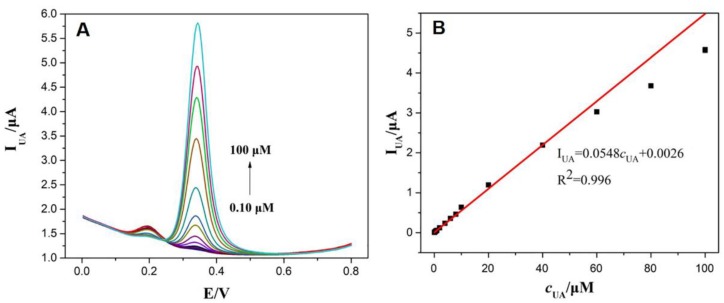
(**A**) DPVs on the d-Fe_2_O_3_/GO/GCE in 0.1 M PBS (pH 5.65) containing 1 μM DA and various concentrations of UA from 0.1 to 100 μM; (**B**) The linear plot of the anodic peak currents of UA versus the UA concentrations varying from 0.1 to 100 μM.

For the individual detection of DA and UA on the d-Fe_2_O_3_/GO/GCE, DPVs were performed in the potential range from 0–0.8 V in 0.1 M PBS (pH 5.65). In this case, only the concentrations of the target species were changed, while the concentrations of the other species remained unaltered. As plotted in Figure 9, the response anodic peak currents of DA increases linearly with the DA concentrations increasing from 0.04 to 4 μM. However, the response anodic peak currents of DA are highly correlation to Napierian logarithm of DA concentrations (ln*C*_DA_) at higher concentration domain (4–100 μM). For individual detection of UA, the response anodic peak currents of UA are in proportion to the all working concentrations from 0.1 to 100 μM (Figure 10). The limit of detection (LOD) for the individual detection of DA and UA are estimated to 4.8 and 12 nM at S/N = 3, respectively. It is noteworthy that addition of the target species does not have obvious interference on the electrochemical responses (i.e., the anodic peak current and potential) of the other species. The results strongly imply that DA and UA can be sensitively and selectively detected on d-Fe_2_O_3_/GO/GCE in the DA and UA mixture (specific concentrations (*C*_DA_/*C*_UA_) range of 0.01–100).

**Figure 11:**
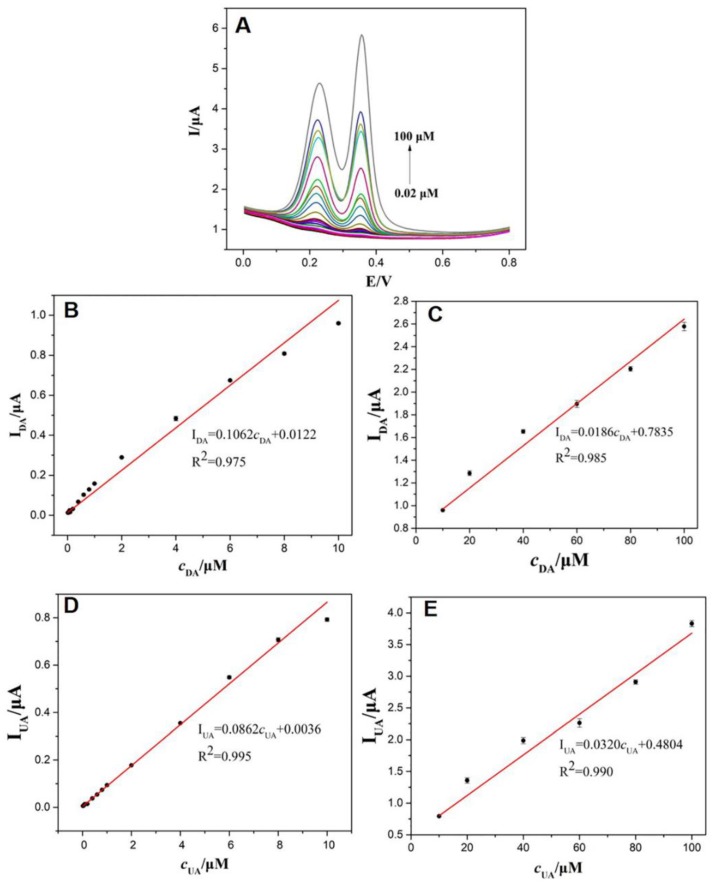
(**A**) DPVs on the d-Fe_2_O_3_/GO/GCE in 0.1 M PBS (pH 5.65) containing different concentrations of DA and UA ranging from 0.02–100 μM; Plots of the anodic peak currents as the function of DA concentrations in the range of 0.02–10 μM (**B**) and 10–100 μM (**C**); Plots of the anodic peak currents as the function of UA concentrations in the range of 0.02–10 μM (**D**) and 10–100 μM (**E**).

Superior electrocatalytic activity of d-Fe_2_O_3_/GO also showed simultaneous detection of DA and UA using DPV technique in 0.1 M PBS (pH 5.65) (Figure 11). Two well-separated anodic peaks relating to the electrooxidation of DA and UA occur on DPV curves using d-Fe_2_O_3_/GO/GCE. Moreover, DPV responses are resolved into two peaks at 0.22 and 0.36 V, which can be attributed to the oxidations of DA and UA, respectively. These results indicating that simultaneous distinguish from the two substances is feasible in mixture solutions. As expected, all anodic peak currents increase linearly with increasing concentrations of DA and UA. Two linear response regions for both DA and UA are obtained within concentration ranges of 0.02–10 μM and 10–100 μM, respectively (Figure 11B–E). The linear regression equations for DA can be expressed as *I*_DA_(μM) = 0.1062*C*_DA_ (μM) + 0.0122 (*R*^2^ = 0.975) and *I*_DA_(μM) = 0.0186*C*_DA_ (μM) + 0.7835 (*R*^2^ = 0.985). The linear regression equations for UA are *I*_UA_(μM) = 0.0862*C*_UA_ (μM) + 0.0036 and *I*_UA_(μM) = 0.0320*C*_DA_(μM) + 0.4804, with correlation coefficient of 0.995 and 0.990, respectively. The LODs are calculated as 3.2 and 2.5 nM for DA and UA, respectively. All results demonstrate that the proposed d-Fe_2_O_3_/GO/GCEs feature wider linear response ranges and lower LOD for the electrochemical oxidation of DA and UA. Hence, the simultaneous detection of DA and UA can be realized on d-Fe_2_O_3_/GO/GCE with high sensitivity and good selectivity. The sensing performances are compared to those in previous reports (Table 1). Clearly, the sensing parameters (i.e., linear response ranges and LOD) of the proposed sensor are comparable to, or even better than most previous reported modified electrodes [7,55,60,61,62,63,64,65,66,67,68,69,70]. The high sensitivity of our proposed sensor is closely related to the synergistic electrocatalytic effect from the d-Fe_2_O_3_ and GO.

### 3.7. Practical Application

**Figure 12:**
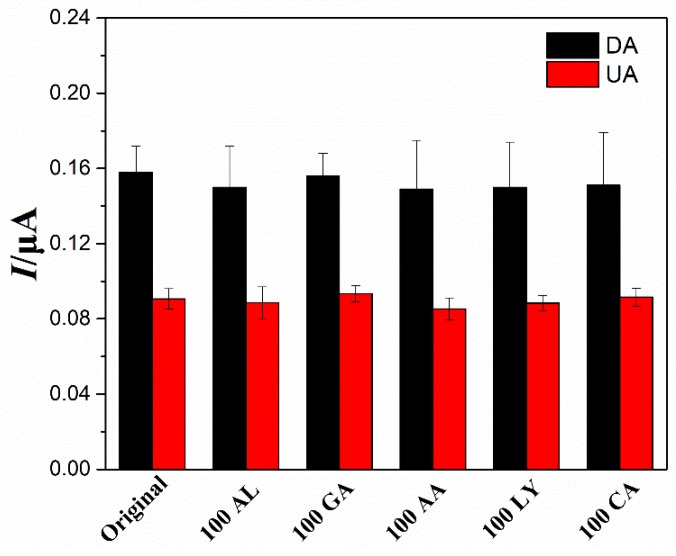
The anodic peak currents of 1 μM DA and UA in the presence of 100-fold alanine (AL), glutamic acid (GA), ascorbic acid (AA), lysine (LY) and citric acid (CA).

Prior to the simultaneous determination of DA and UA in actual samples, the selectivity, repeatability, stability and reproducibility were also investigated. In order to assess the anti-interference of the proposed d-Fe_2_O_3_/GO/GCE toward the DA and UA, the DPV responses of the DA and UA in the presence of potential interfering species were recorded. Negligible interference with accepted relative error (less than 5.70%) even in the presence of 100-fold ascorbic acid, citric acid, alanine, glutamic acid, and lysine (Figure 12). To assess electrode reproducibility, the variation on anodic peak currents in the 1 μM DA and UA (1:1) mixture solution was measured at room temperature using five d-Fe_2_O_3_/GO/GCE, which were fabricated by the same procedure. The relative standard deviations (RSD) for the anodic peak current are 4.56% and 3.03%, respectively, indicating that the electrode fabrication has high reproducibility. To evaluate the repeatability, seven successive measurements for detection of 1 μM DA and UA (1:1) were carried out. The RSD for DA and UA are 5.07% and 4.81%, respectively, suggesting good repeatability.

To verify applicability, the concentrations of DA and UA in actuate samples were also detected on d-Fe_2_O_3_/GO/GCE. Since DA and UA always coexist in most biological fluids, the practicability of d-Fe_2_O_3_/GO/GCE in actual sample detection was assessed with human urine and serum samples. The determined results listed in Table 2 were carefully estimated from the standard curves. To further validate the accuracy and precision of the proposed sensor, a series of known concentration solutions of DA and UA were spiked to the actual samples to calculate the recovery. The recoveries of 94.1–106.4% and 95.2–108.2% are obtained for DA and UA, respectively. DPVs for the analysis of human serum samples with 100-fold dilution are presented in Figure S1. Notably, the shape and position of the DPVs are not affected when using real samples. These results confirm that the biological matrixes such as human urine and serum does not affect the simultaneous determination of DA and UA.

## 4. Conclusions

In summary, the influence of Fe_2_O_3_/GO morphologies on electrochemical sensing performances was studied systematically. Cubic, thorhombic and discal Fe_2_O_3_ NPs with a uniform size and controllable structure were successfully prepared by a facile meta-ion mediated hydrothermal route. When coupled with GO nanosheets and then worked as sensing films for the simultaneous detection of DA and UA, the α-Fe_2_O_3_ NPs with discal morphology displayed the highest electrocatalytic activity. This remarkable electrocatalytic behavior was highly correlated with the discal shapes with more surface defects and a rougher surface. The synergistic effect from d-Fe_2_O_3_ NPs and GO nanosheets contributed to a significant enhancement in the response currents. As a consequence, two wide detection ranges (0.02–10 μM and 10–100 μM) were obtained for DA and UA, with very low limit of detection (LOD) of 3.2 and 2.5 nM for DA and UA, respectively. Moreover, the d-Fe_2_O_3_/GO nanohybrids showed good selectivity and reproducibility. The proposed d-Fe_2_O_3_/GO nanohybrids have become one of the most competitive candidates as sensing materials for the simultaneous determination of DA and UA in various real samples.

## Figures and Tables

**Table 1:** Comparison analytical performance between previous reports and the proposed d-Fe_2_O_3_/GO/GCE for the simultaneous detection of DA and UA.

Electrodes	Methods	Detection Range (μM)		LOD (μM)		Ref.
		DA	UA	DA	UA	
Au/Cu_2_O/rGO/GCE	DPV	10–90	100–900	3.9	6.5	[7]
NSG–Fe_2_O_3_/GCE	DPV	0.3–210		0.035		[55]
Pd/RGO/GCE	DPV	0.45–71	6–469.5	0.18	1.6	[60]
Pt/RGO/GCE	DPV	10–170	10–130	0.25	0.45	[61]
ZnO/SPCE	DPV	0.1–374	0.1–169	0.004	0.00849	[62]
Fe_3_O_4_/rGO/GCE	DPV	0.5–100		0.12		[63]
AuPtNPs/S-NS-GR/GCE	DPV	0.01–400	1–1000	0.006	0.0038	[64]
Au–Pt/GO–ERGO	DPV	0.0682–49,800	0.125–82,800	0.0207	0.0407	[65]
Fe_2_O_3_/NrGO/GCE	Amperometry	0.5–340		0.49		[66]
ZnO/PANI/rGO/GCE	DPV	0.1–90	0.5–90	0.017	0.12	[67]
pCu_2_O NS-rGO/GCE	DPV	0.05–109	1–138	0.015	0.112	[68]
Zn-NiAl LDH/rGO/GCE	DPV	0.0001–1	0.0011–0.95	0.0001	0.0009	[69]
α-Fe_2_O_3_@Au-Pd/GCE	SWV	0.1–1000	1–1000	0.0000138	0.97	[70]
d-Fe_2_O_3_/GO/GCE	DPV	0.02–10; 10–100	0.02–10; 10–100	0.0032	0.0025	This work

**Table 2:** Detection results of DA and UA in human serum and urine samples using d-Fe_2_O_3_/GO/GCE.

Samples ^a^		Detected (μM)	Added (μM)	Found (μM)	RSD (%)	Recovery (%)
Serum	DA	ND ^b^	20	18.82	3.75	94.1
			40	38.26	2.86	95.7
	UA	20.6	20	41.76	2.75	105.8
			40	63.86	2.21	108.2
Urine	DA	ND ^b^	20	21.06	4.98	105.3
			40	42.56	3.67	106.4
	UA	35.24	20	54.27	2.62	95.2
			40	74.52	1.39	98.2

^a^ The human urine and serum samples were detected at 100-fold and 10-fold dilution, respectively. ^b^ Not detected.

## Supplementary Materials

The following are available online at https://www.mdpi.com/2079-4991/9/6/835/s1, Figure S1: DPVs for the analysis of human serum samples with 100-fold dilution.
