# Supplementary material for: Morphology–Dependent Electrochemical Sensing Properties of Iron Oxide–Graphene Oxide Nanohybrids for Dopamine and Uric Acid
Source: Nanomaterials (Basel). 2019 Jun 1;9(6):835. doi: 10.3390/nano9060835 (PMC6631868; doi:10.3390/nano9060835)
Supplement: Supplementary file 1 [file nanomaterials-09-00835-s001.pdf]

Supporting Information

Morphology–Dependent Electrochemical Sensing Properties of Iron Oxide–Graphene Oxide Nanohybrids for Dopamine and Uric Acid

Zhaotian Cai [1,†], Yabing Ye [1,†], Xuan Wan [1], Jun Liu [1], Shihui Yang [1], Yonghui Xia [2], Guangli Li [1,*] and Quanguo He [1,*]

1 Hunan Key Laboratory of Biomedical Nanomaterials and Devices, College of Life Sciences and Chemistry, Hunan University of Technology, Zhuzhou 412007, China; caizhaotian1998@163.com (Z.C.); yyb980501@163.com (Y.Y.); wanxuan1111@163.com (X.W.); liu.jun.1015@163.com (J.L.); yangshihui0522@163.com (S.Y.)

2 Zhuzhou Institute for Food and Drug Control, Zhuzhou 412000, China; Sunnyxia0710@163.com

* Correspondence: guangli010@hut.edu.cn (G.L.); hequanguo@hut.edu.cn (Q.H.); Tel.: +86-0731-2218-3382 (G.L.&Q.H.)

† These authors contributed equally to this work.

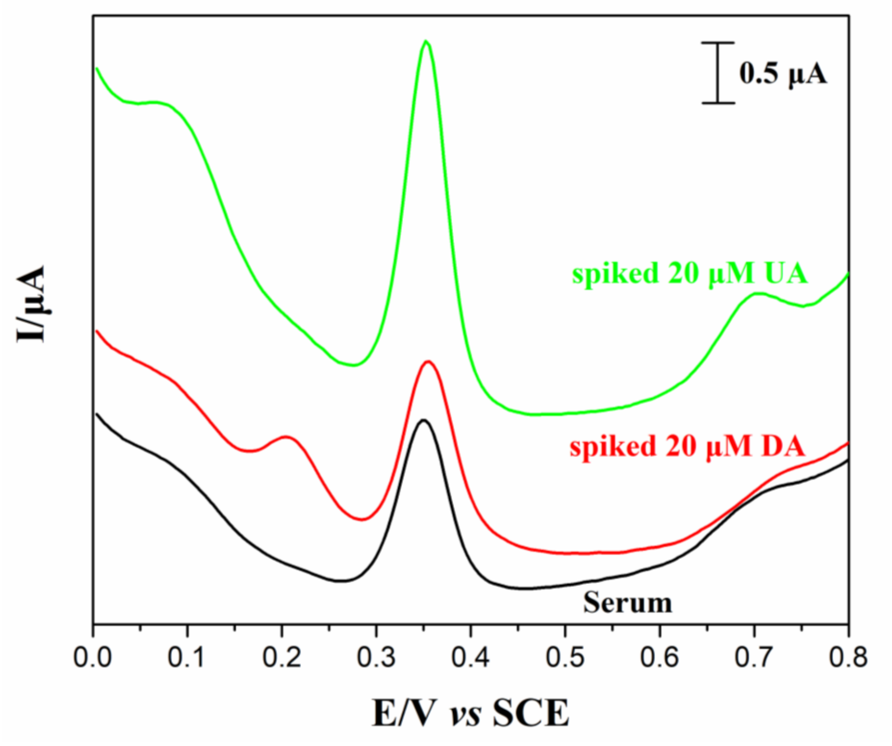

Figure S1. DPVs for the analysis of human serum samples with 100-fold dilution.
